# Relationship between Selected Trace Elements in Human Milk and Psychosocial Characteristics in Korean Early Postpartum Women

**DOI:** 10.3390/ijerph18010350

**Published:** 2021-01-05

**Authors:** Sookjin Noh, Eunjoo Lee

**Affiliations:** Department of Nursing, Kyungnam University, Changwon 51767, Korea; waa00@naver.com

**Keywords:** postpartum, human milk, calcium, sodium, iron, selenium

## Abstract

This study aimed to identify the relationship between selected trace elements in human milk and depression, breastfeeding self-efficacy, sleep satisfaction, and the demographic and obstetric characteristics of lactating mothers. Forty lactating mothers recovering after birth in two postpartum care centers located in Changwon, South Korea, were studied. Calcium, sodium, iron, and selenium concentrations in human milk were analyzed using inductively coupled plasma mass spectrometry at the analysis center of Kyungnam University. Data were analyzed using descriptive statistics, the Kruskal-Wallis test, the Mann-Whiney U test, and Spearman’s correlation coefficients using an SPSS 23.0 program. There were no significant differences in concentration of selected trace elements (calcium, sodium, iron, and selenium) in human milk according to demographic and obstetric characteristics. Sodium concentration was negatively correlated with breastfeeding self-efficacy (r = −0.51, CI = −0.71 to −0.24). Selenium concentration was negatively correlated with calcium concentration (r = −0.34, CI = −0.58 to 0.38). Postpartum depression was negatively correlated with sleep satisfaction (r = −0.46, CI = −0.67 to −0.17) and with breastfeeding self-efficacy (r = −0.41, CI = −0.64 to −0.11). Breastfeeding self-efficacy was positively correlated with sleep satisfaction (r = 0.48, CI = 0.20 to 0.69). This study suggests that sodium concentration in human milk is associated with breastfeeding self-efficacy.

## 1. Introduction

The birth rate for South Korea in 2018 was 0.977 births per 1000 people, which was the lowest rate in the world for the past three years [1]. The average age of pregnant women is gradually increasing, from 28.1 in 1996 to 32.8 in 2017 [1]. The current trends of avoiding marriage or childbirth can cause an increase of mother’s age and low rates of breastfeeding [2].

The World Health Organization (WHO) recommends exclusive breastfeeding for the first six months [3]. However, a report from the Korean ministry of health and welfare for postpartum care showed that the rates of exclusive breastfeeding were 33.8% in the first week after birth and 8.8% for infants less than six months of age, which do not meet the WHO guidelines [4].

Human milk contains proteins, sugars, and fats that are essential for the health of infants. It also contains immune-enhancing ingredients effective in preventing infectious diseases, gastrointestinal disorders, eczema, and allergic diseases [5]. Breastfeeding helps infants to become emotionally stable and develop their teeth and mandible [6]. Human milk helps to notably strengthen an infant’s immune system as well as promote growth and development [7]. Zinc and various hormones, enzymes, and nutrients in breastmilk are important for protein and nucleic acid homeostasis [8]. Iron is required not only for hemopoiesis, but also for Fe-S cluster synthesis, which is especially important for respiration and DNA replication [9]. Calcium is the main component of animal bones and teeth, and calcium ions are involved in regulatory functions [10]. Human milk has lower levels of trace elements compared to cow’s milk, but its utilization in the human body is higher [11]. Before birth, the fetus receives a sufficient amount of trace elements through the placenta, but after birth, elements are absorbed in the gastrointestinal tract by ingestion [12]. Therefore, breastfed babies have more trace elements than formula-fed infants.

The trace elements in human milk are affected by the maternal characteristics containing nutritional status, age, presence of disease, current neonatal period [13], drinking, smoking, and consumption of dietary supplements [14]. The health status, depression [15], stress, and early removal of colostrum [16] may also influence the amount, composition and quality of human milk. In other words, a various of characteristics of the mothers are related with milk levels of trace elements.

The early postpartum period is a sensitive period for acquiring and adapting to the postpartum maternal role and is a high risk of stopping breastfeeding due to physical and psychological changes [16]. Recently, most mothers tend to stay in postpartum care centers in South Korea, which increases the separation time from their babies and reduces opportunities for breastfeeding [17]. In addition, since the trace elements and nutrients of breast milk decrease over time after childbirth [14], human milk should be delivered to the fetus during the early postpartum period.

Previous studies on trace elements in human milk included factors affecting its trace elements and immune substances [14], longitudinal changes in trace elements [18], zinc and copper concentration [19], zinc in plasma and breast [20], maternal diet and selenium [21]. Most studies have focused on component analysis or nutritional aspects of human milk, but only a few studies have confirmed the relationship between trace elements in human milk and psychosocial variables [22,23].

Therefore, this study aims to investigate the relationships among demographic and psychosocial characteristics and, selected trace elements in human milk in early postpartum women. The results of this study can provide basic data for programs to promote breastfeeding and further contribute to improving the practice and awareness of breastfeeding for postnatal women.

## 2. Material and Methods

### 2.1. Setting and Sample

This was a descriptive research to confirm the relationship between selected trace elements in human milk and psychosocial variables in lactating mothers during early postpartum period. The participants recruited were 40 lactating mothers admitted to postpartum care centers at two women’s hospitals located in Changwon, South Korea. The recruitment notice was displayed at the hospitals’ cafeterias. We obtained written consent from all participants, who voluntarily consented to participate in this study. 

Participants met the following inclusion criteria: Mothers who (1) were breastfeeding within seven days after birth, (2) were ≥20 years old, (3) delivered a healthy baby with an APGAR (appearance pulse grimace activity respiration) score ≥ 8 and who weighted > 2500 g at birth between 37 and 42 weeks, and (4) agreed to voluntarily participate in the study, with understanding of the purpose of the study.

Exclusion criteria were mothers that were diagnosed with mental illnesses such as depression and bipolar disorder, as well as those who were unable to breastfeed for health reasons. Sample size was determined using G-power 3.2.1 software; for a correlation analysis with an effect size of 0.45, a significance level of 0.05, and a power of 0.85, the minimum required sample size was 38. The sample size in previous studies that analyzed human milk contents ranged from 17 to 30 [24,25].

### 2.2. Procedure

Data were collected from 20 August 2019 to 20 September 2019. The study was approved by the Institutional Review Board of Kyungnam University in Changwon, Korea (1040460-A-2019-041). This study began after obtaining permission from the heads of the postpartum care center at the two hospitals. On the seventh day after delivery, those who consented to participate in the study responded to questionnaires that assessed a variety of demographic characteristics, postpartum depression, breastfeeding self-efficacy, and sleep satisfaction. 

Women were instructed to collect from remaining milk with 5 mL after the first feeding by pumping on the morning of the seventh day after birth, use the plastic tubes, and freeze the milk until the staff arrived later that day. Before milk sample collection, the nipples and areolas of the breasts were cleaned with wet gauze. Samples were transported on ice and then stored at −20 °C.

The selected trace elements (calcium, sodium, iron, and selenium) in human milk were conducted at the Kyungnam University analysis center with ICP MS NEXION 350 D (Perkin Elmer, Waltham, MA, USA) which is a type of mass spectrometry that detects the emission line generated by exciting the electrons of elements in the sample. It is used as a light source of plasma coupled to electromagnetic fields inducted by a coil through which electric current flows and then measured by the emission line generated through the transition until the electrons of the sample elements float. The apparatus consists of a light source, a radiofrequency generator, a torch, an induction coil, and an atomization device. A 1.0 mL amount of each sample was mixed with 5 mL of 65% HNO_3._

### 2.3. Measurements

#### 2.3.1. Demographic Characteristics

Demographic characteristics included maternal age, education, religion, monthly household income, smoking, drinking frequency of alcoholic beverages, history of allergy (atopic dermatitis, allergic rhinitis, asthma and food, air, dust and animal hair allergy), health problem (cardiovascular, respiratory, gastrointestinal and musculoskeletal disease), and types of families (a nuclear family or an extended family). Obstetrical characteristics included parity (primiparous or multiparous), planned pregnancy, regular exercise (more than once a week or not), feeding plan (exclusive breastfeeding or mixed feeding), depression, use of nutritional supplements, and weight gain during pregnancy.

#### 2.3.2. Postpartum Depression

Postpartum depression was measured using the Korean version of the Edinburgh Postpartum Depression Scale (EPDS), which was originally developed by Cox, Holden, and Sagovsky [26] and translated into a Korean version by Kim [27]. This tool is a set of 10 screening questions that measure whether postpartum women have symptoms that are common in women with depression. Women select the number that comes closest to how they have felt during the past seven days. An example from this questionnaire is as follows: “I have been able to laugh and see the funny side of things”. Each item is rated on a four-point Likert scale from 0 (strongly disagree) to 3 (strongly agree). The total scores range from 0 to 30, with higher scores indicating high postpartum depression. The Cronbach’s ⍺ was 0.87 in Cox et al.’s study [26], 0.84 in Kim’s study [27], and 0.91 in this study.

#### 2.3.3. Breastfeeding Self-Efficacy

A short form breastfeeding self-efficacy scale was used to assess breastfeeding self-efficacy at baseline for postpartum women. It was developed by Dennis [28] and translated into a Korean version by Kim [29]. It is a 14-item self-reporting measurement. An example of this questionnaire is as follows: ”Determine that my baby is getting enough milk”. All items are phrased as “I can always” and rated on a five-point Likert scale, with 1 meaning “not at all confident” and 5 meaning “always confident”. The total scores range from 14 to 70, with higher scores indicating high breastfeeding self-efficacy. The Cronbach’s ⍺ was 0.96 in Dennis’s study [28], 0.92 in Kim’s study [29], and 0.89 in this study. 

#### 2.3.4. Sleep Satisfaction

Sleep satisfaction was measured using the sleep satisfaction scale developed by Song [30]. This tool consists of two questions regarding overall sleep satisfaction and sleep disturbance from baby care. Sleep satisfaction is determined using one question: “how much is your sleep satisfaction overall?”. It is rated on an 11-point Likert scale from 0 (not resting, not feeling well) to 10 (feel lighter), with higher scores indicating high sleep satisfaction. Sleep disturbance is determined using one question: “how much is your sleep disturbed by taking care of your baby overall?”. It is rated on an 11-point Likert scale from 0 (not disturbed) to 10 (very disturbed), with higher scores indicating high sleep disturbance. The Cronbach’s ⍺ was 0.84 in Song’s study [30] and 0.88 in this study.

### 2.4. Data Analysis

The collected data were analyzed using the SPSS statistical software for WIN, ver. 23.0 (IBM, Armonk, NY, USA). Data were checked for normality of variables using the Shapiro-Wilk test. Non-normally distributed variables are described as the median and interquartile range (IQR). Differences in selected trace elements of human milk according to demographic and obstetrical characteristics were analyzed by the Mann-Whitney U test or Kruskal-Wallis test for non-parametric data. Spearman’s correlation coefficient was used to measure the association among variables. A statistical significance level was set at 0.05 and the p-value presented was two-tailed.

## 3. Results

### 3.1. Demographic and Obstetrical Characteristics

Demographic and obstetrical characteristics of subjects are presented in Table 1. The median age (IQR) of lactating mothers was 31 (6.5) years. A total of 17.5% of the participants were high school graduates or lower and 82.5% were college graduates or higher. Half of the participants had a religion. The highest rate of monthly household income was 4.51 million KRW or higher (35.0%), and 40% reported drinking alcoholic beverages 1–2 times per month. 80% of mothers were not smokers and 82.5% reported not having any health problem at the time of the study interview.

A total of 57.5% of the participants had a vaginal delivery. The median gestational age (IQR) was 39 (1) weeks. Just over half (57.5%) of mothers were having their first baby and 52.5% of infants were male. The median weight gain during pregnancy (IQR) was 12 (6) kg. The majority of participants had planned the pregnancy (87.5%). Of the participants, 65.0% did not regularly exercise during pregnancy. Only 13 mothers had a plan to do exclusive breastfeeding. All participants did not experience depression during pregnancy. Nutritional supplements were ingested by 87.5% participants. 

### 3.2. Postpartum Depression, Breastfeeding Self-Efficacy, and Sleep Satisfaction

The median postpartum depression, breastfeeding self-efficacy, and sleep satisfaction are presented in Table 2. The median (Md) postpartum depression score (IQR) was 6 (2.75), the median breastfeeding self-efficacy score was 41.5 (11.75), and the median sleep satisfaction score was 5 (17.5).

### 3.3. Selected Trace Elements’ Concentrations in Human Milk

The selected trace elements’ concentrations in human milk are presented in Table 3. The median calcium concentration (IQR) of lactating mothers was 120.4 (75.8) mg/L, median sodium concentration was 134.3 (108.4) mg/L, median iron concentration was 0.7 (0.6) mg/L, and median selenium concentration was 1.0 (0.9) mg/L.

### 3.4. Differences in Selected Trace Elements’ Concentrations in Human Milk According to Demographic Characteristics

The differences in selected trace elements’ concentrations in human milk according to demographic characteristics are presented in Table 4. There were no significant (*p* > 0.05) differences in selected trace elements’ (calcium, sodium, iron, and selenium) concentrations in human milk according to demographic characteristics.

### 3.5. Differences in Selected Trace Elements’ Concentrations in Human Milk According to Obstetrical Characteristics

The differences in selected trace elements’ concentrations in human milk according to obstetrics characteristics are presented in Table 5. There were no significant (*p* > 0.05) differences in selected trace elements’ (calcium, sodium, iron, and selenium) concentrations in human milk according to obstetric characteristics.

### 3.6. Correlations between Psychosocial Characteristics and Selected Trace Elements in Human Milk

The correlations between psychosocial characteristics and selected trace elements in human milk are presented in Table 6. Sodium concentration was negatively correlated with breastfeeding self-efficacy (r = −0.51, CI = −0.71 to −0.24). Selenium concentration was negatively correlated with calcium concentration (r = −0.34, CI = −0.58 to 0.03). Postpartum depression was negatively correlated with sleep satisfaction (r = −0.46, CI = −0.67 to −0.17) and with breastfeeding self-efficacy (r = −0.41, CI = −0.64 to −0.11) Breastfeeding self-efficacy was positively correlated with sleep satisfaction (r = 0 .48, CI = 0.20 to 0.69).

## 4. Discussion

The composition of breast milk varies with the time passed after birth, during nursing, and with maternal nutrition [31]. As the baby grows, the amount or composition of breast milk increases in accordance with its needs. But at 4–6 months, it is difficult to meet all the nutritional needs of the baby with only breast milk, so supplementary diets or supplements are needed [31]. The nutritional status of lactating mothers is important because it may influence the nutrient concentration of human milk; however, maintaining the nutrients in human milk can deplete the mother’s body reservoir [31]. The maternal diet during pregnancy is related to the supply of fatty acids during lactation, and arachidonic acid (AA) and docosahexaenoic acid (DHA) provided by the mother to the fetus and infant are directly related to the mother’s dietary intake and body storage [32]. Maternal dietary intake and some micronutrients, including fat soluble vitamins, vitamin B1, and vitamin C, are also associated with breast milk composition [33]. Thus, the components of breastfeeding can be affected by various factors. This study attempted to verify the relationships among the selected trace elements in human milk, psychosocial characteristics (depression, breastfeeding self-efficacy, and sleep satisfaction), and the demographic and obstetric characteristics of lactating mothers.

### 4.1. Differences in Selected Trace Elements’ Concentrations in Human Milk According to Demographic and Obstetric Characteristics

There were no significant (*p* > 0.05) differences in selected trace elements’ (calcium, sodium, iron, and selenium) concentrations in human milk according to demographic and obstetric characteristics.

While the calcium concentration of a female baby was higher than a male baby, it was not statistically significant. In Kwon et al.’ [14] also showed no differences in calcium concentration between male and female infants. A few country studies [23,34] revealed that the calcium concentration in human milk differed from infant sex. In the case of Korean women, there was no differences in calcium levels according to infant sex because of insufficient calcium intake and low levels of calcium in human milk.

The sodium concentration was lower in mothers engaging in regular exercise than irregular exercise, though it was not statistically significant. Regular exercise during pregnancy seems to lower sodium concentration in human milk. Exercise seems to help in the balance of water and electrolytes in the body [35], especially the secretion of sodium, which leads to lower the sodium concentration in the vessel [36]. Elevated sodium concentration in vessels increases osmotic concentration, which increases water and blood volume and disturbs electrolyte homeostasis [37]. In lactating mothers, a high sodium concentration in human milk impairs milk release [38], which can lead to problems such as mastitis, breast pain, and breast engorgement [39]. The relationship between sodium concentration in human milk and exercise during pregnancy should be studied more clearly.

Iron was not significantly different from the various characteristics in previous studies [14,24,40]. However, a few studies showed that the iron content in breast milk donated to breast milk banks had a statistically significant difference in accordance with the status of maternal smoking [41]. In the secondary analysis study, the serum ferritin concentration of smokers also was significantly higher than that of non-smokers [42]. In addition, newborns born to mothers who smoked during pregnancy had significantly higher carbon monoxide hemoglobin, which causes fetal hypoxia at birth [43]. Thus, there have been several prior studies showing a relationship between iron and mothers’ smoking. Regardless of the results of this study, it is well known that smoking has negative consequences on the growth and development of the fetus. Therefore, it should be considered as important in prenatal education for lactating women. 

Few studies have confirmed the relationship between selenium in breast milk and maternal characteristics. The result reported that the lower the selenium concentration collected from the toenails during pregnancy, the more severe the symptoms of preeclampsia [44]. In addition, a study on mothers in the UK found that the lower the concentration of selenium collected from the toenail, the higher the risk of developing gestational hypertension [45].

### 4.2. Correlations between Psychosocial Characteristics and Selected Trace Elements in Human Milk

#### 4.2.1. Correlations between Psychosocial Characteristics and Selected Trace Elements in Human Milk

The sodium concentration in human milk was negatively correlated with breastfeeding self-efficacy. Breastfeeding self-efficacy refers to the mother’s confidence in her ability to engage in actions required for breastfeeding [46]. Women with higher breastfeeding self-efficacy engage more actively in breastfeeding and are cautious about sodium intake, which can lower the sodium concentration in breast milk [47]. In previous studies [16,38], sodium concentration was reported to be highly related to breast congestion. If breastfeeding is not effectively achieved, breast problems such as breast infection, breast pain or tenderness, and breast lumps can be caused by milk stagnation or high levels of sodium in human milk [48]. Lack of breastfeeding after childbirth can lead to hypernatremia in the fetus [49]. Since the sodium concentration in breast milk decreases as the amount of milk secretion increases or as the birth time goes on [16]. Failure of early discharge of colostrum from the breast can cause high levels of sodium concentration. [16]. Thus, the sodium concentration in human milk will be higher in women who are not active in breastfeeding in the early postpartum period. Education to straighten effective breastfeeding behaviors such as massage, dietary intake, and good posture for breastfeeding should be provided to pregnant women.

#### 4.2.2. Correlations among Psychosocial Characteristics

Breastfeeding self-efficacy was significantly negatively correlated with postpartum depression. Lee and Cho’s study [50] also showed that breastfeeding self-efficacy influenced postpartum depression. Mothers with high breastfeeding self-efficacy had lower postpartum depression and higher exclusive breastfeeding rates than mothers with low breastfeeding self-efficacy [51]. Increasing breastfeeding self-efficacy would help prevent postpartum depression and increase the breastfeeding rate.

#### 4.2.3. Correlations among the Selected Trace Elements

Selenium concentration in human milk was negatively correlated with calcium levels. This means that the higher the concentration of selenium, the lower the concentration of calcium. Selenium plays a role in alleviating muscle pain and halting the progression of myasthenia gravis and muscle weakening [52]. The symptoms of selenium deficiency in human included muscle wasting, and chronic diseases such as arthritis and cardiovascular disease [53]. The results of previous studies [54,55] showed that hypercalcemia exhibits symptoms of muscle soreness, general weakness, and muscle weakness. Through this, the inverse relationship between selenium and calcium concentration can be inferred. The selenium requirement in women is increased during lactation due to the amount of selenium that is secreted with breast milk while feeding the infant [56]. Meanwhile, elevated selenium intake has a negative effect on bone mass as calcium intake is low [57]. Therefore, lactating mothers should need to maintain a balance between calcium and selenium intake [58].

## 5. Conclusions

The results of this study are meaningful in that it is found that the higher the breastfeeding self-efficacy, the lower the sodium concentration in breast milk. The results suggest that enhancing breastfeeding self-efficacy can reduce sodium concentration in human milk and prevent breast congestion. Therefore, lactating mothers should have confidence in breastfeeding and actively practice breastfeeding behaviors. They also should pay attention to their intake, considering that calcium and selenium concentration can affect each other.

There are some limitations in this study. First, the study sample did not reflect the general population because this study had limited data to gain access to the cultural background, geographic scope, and socioeconomic status of patients because samples were collected from only two hospitals. Second, it might be difficult to make definitive conclusions about the association between dependent and independent variables using a small sample size. Third, we did not consider the potential confounders that might have affected the trace element status in human milk. Further studies need to be carried out using larger and various samples and controlling for confounding variables in order to identify the results.

## Figures and Tables

**Table 1:** Demographic and obstetrical characteristics of subjects (N = 40).

Characteristic	Categories	Median (IQR ^1^), n (%)
Age (years)		31 (6.5)
Education	High school graduate or less	7 (17.5)
	More than college graduate	33 (82.5)
Religion	Yes	20 (50.0)
	No ^2^	20 (50.0)
Monthly household income (10,000 KRW ^3^)	250≥	5 (12.5)
	251~350	11 (27.5)
	351~450	10 (25.0)
	450<	14 (35.0)
Drinking frequency of alcoholic beverages	None	13 (32.5)
	1~2/a month	16 (40.0)
	1~2/a week	8 (20.0)
	3~4/a week	3 (7.5)
Smoking	Yes	8 (20.0)
	No	32 (80.0)
History of allergy ^4^	Yes	9 (22.5)
	No	31 (77.5)
Health problem ^5^	Yes	7 (17.5)
	No	33 (82.5)
Types of families	A nuclear family ^6^	33 (82.5)
	An extended family ^7^	7 (17.5)
Method of delivery	Vaginal delivery	23 (57.5)
	Cesarean section	17 (42.5)
Gestation week		39 (1)
Parity	Primiparous	23 (57.5)
	Multiparous	17 (42.5)
Planned pregnancy	Yes	35 (87.5)
	No	5 (12.5)
Regular exercise ^8^	Yes	14 (35.0)
	No	26 (65.0)
Breastfeeding plan	Exclusive breastfeeding	13 (32.5)
	Mixed feeding	27 (67.5)
Depression during pregnancy	Yes	0 (0.0)
	No	40 (100.0)
Use of nutritional supplements	Yes	35 (87.5)
	No	5 (12.5)
Weight gain during pregnancy (kg)		12 (6)
Infant’s sex	Male	21 (52.5)
	Female	19 (47.5)
Infant’s birth weight (kg)		3.3 (0.5)

Notes: ^1^ IQR; interquartile range. ^2^ Religion no; atheists. ^3^ KRW; Korean won. ^4^ History of allergy; atopic dermatitis, allergic rhinitis, asthma and food, air, dust and animal hair allergy). ^5^ Health problem; cardiovascular, respiratory, gastro-intestinal and musculoskeletal disease. ^6^ A nuclear family; a family consisting of two parents and their children. ^7^ An extended family; a family that extends beyond the nuclear family, including grandparents, uncles, and other relatives. ^8^ Regular exercise; more than once a week.

**Table 2:** Postpartum depression, breastfeeding self-efficacy, and sleep satisfaction (N = 40).

Variables	Minimum	Maximum	Median	IQR ^1^
Postpartum depression	0	19	6	2.75
Breastfeeding self-efficacy	25	63	41.5	11.75
Sleep satisfaction	1	16	5	17.5

Notes: ^1^ IQR; interquartile range.

**Table 3:** Selected trace elements’ concentrations in human milk (N = 40).

Trace Elements	Minimum	Maximum	Median	IQR ^1^
Ca ^2^ mg/L	72.21	313.80	120.4	75.8
Na ^3^ mg/L	62.52	372.80	134.3	108.4
Fe ^4^ mg/L	0.00	2.87	0.7	0.6
Se ^5^ mg/L	0.00	0.04	1.0	0.9

Notes: ^1^ IQR; interquartile range. ^2^ Ca; calcium. ^3^ Na; sodium. ^4^ Fe; iron. ^5^ Se; selenium.

**Table 4:** Differences in selected trace elements’ concentrations in human milk according to demographic characteristics (N = 40).

Characteristics	Categories	Ca ^1^		Na ^2^		Fe ^3^		Se ^4^	
		U/*x*^2^	*p*	U/*x*^2^	*p*	U/*x*^2^	*p*	U/*x*^2^	*p*
Education ^a^	High school graduate or less	96.00	0.488	87.00	0.310	97.00	0.510	108.00	0.789
	More than college graduates								
Religion ^a^	No	196.00	0.914	174.00	0.482	185.00	0.685	187.00	0.725
	Yes								
Monthly household income ^b^	250≥	2.98	0.394	0.66	0.882	2.85	0.415	1.05	0.788
	251~350								
	351~450								
	450<								
Drinking frequency of alcoholic beverages ^b^	None	4.32	0.228	2.76	0.430	0.68	0.876	0.87	0.832
	1~2/a month								
	1~2/a week								
	3~4/a week								
Smoking ^a^	Yes	127.50	0.987	106.00	0.457	118.00	0.735	97.00	0.295
	No								
History of allergy ^a^	Yes	107.00	0.300	126.00	0.662	84.00	0.072	111.00	0.356
	No								
Health problem ^a^	Yes	112.50	0.915	103.50	0.669	79.00	0.194	96.00	0.488
	No								
Types of families ^a^	Nuclear	106.00	0.735	104.00	0.682	100.00	0.581	91.00	0.383
	Extended								

Notes: ^1^ Ca; calcium. ^2^ Na; sodium. ^3^ Fe; iron. ^4^ Se; selenium. Data was analyzed by ^a^ Mann-Whitney U test or ^b^ Kruskal-Wallis test.

**Table 5:** Differences in trace elements’ concentrations in human milk according to obstetrical characteristics (N = 40).

Characteristic	Categories	Ca ^1^		Na ^2^		Fe ^3^		Se ^4^	
		U/*x*^2^	*p*	U/*x^2^*	*p*	U/*x*^2^	*p*	U/*x*^2^	*p*
Methods of delivery ^a^	Vaginal	153.00	0.245	190.00	0.820	188.00	0.837	148.00	0.194
	Cesarean section								
Parity ^a^	Primiparous	168.00	0.452	169.00	0.978	169.00	0.468	181.00	0.692
	Multiparous								
Infant’s sex ^a^	Male	129.00	0.056	193.00	0.860	158.00	0.261	186.00	0.715
	Female								
Planned pregnancy ^a^	Yes	73.50	0.567	78.00	0.698	71.00	0.500	46.00	0.090
	No								
Regular exercise ^a^	Yes	150.00	0.372	115.50	0.059	128.00	0.126	131.00	0.148
	No								
Breastfeeding plan ^b^	Exclusive breastfeeding	104.50	0.617	110.00	0.779	89.00	0.262	87.00	0.230
	Mixed feeding								
Use of nutritional supplements ^a^	Yes	52.50	0.152	70.00	0.474	81.00	0.790	67.00	0.402
	No								

Notes: ^1^ Ca; calcium. ^2^ Na; sodium. ^3^ Fe; iron. ^4^ Se; selenium. Data was analyzed by ^a^ Mann-Whitney U test or ^b^ Kruskal -Wallis test.

**Table 6:** The Spearman correlation coefficients (*r*) between psychosocial characteristics and selected trace elements in human milk (N = 40).

Variables	Postpartum Depression	Breastfeeding Self-Efficacy	Sleep Satisfaction	Ca ^1^	Na ^2^	Fe ^3^	Se ^4^
	Spearman’s *r* (95% Confidence Interval)						
Postpartum depression	1	**−0.41** (−0.64~−0.11)	**−0.46** (−0.67~−0.17)	0.21 (−0.10~0.45)	0.07 (−0.24~0.37)	0.09 (−0.22~0.39)	−0.24 (−0.51~0.07)
Breastfeeding self-efficacy		1	**0.48** (0.20~0.69)	−0.17 (−0.45~0.15)	**−0.51** (−0.71~−0.24)	0.04 (−0.27~0.35)	−0.27 (−0.54~0.05)
Sleep satisfaction			1	−0.10 (−0.40~0.21)	−0.09 (−0.40~0.22)	−0.17 (−0.46~0.15)	0.04 (−0.27~0.35)
Ca				1	0.27 (−0.05~0.54)	0.30 (−0.01~0.56)	**−0.34** (−0.58~0.03)
Na					1	−0.10 (−0.4~0.21)	−0.20 (−0.48~0.12)
Fe						1	−0.14 (−0.43~0.18)

Notes: ^1^ Ca; calcium. ^2^ Na; sodium. ^3^ Fe; iron. ^4^ Se; selenium. Significant correlations are indicated in bold. Data show Spearman correlation.

## Data Availability

The data used and/or analyzed during the current study are available from the corresponding author on request.
